# Effects of boiling water treatment on the physical properties of *Quercus variabilis* virgin cork grown in Korea

**DOI:** 10.1038/s41598-024-56110-5

**Published:** 2024-03-05

**Authors:** Denni Prasetia, Byantara Darsan Purusatama, Jong Ho Kim, Jae Hyuk Jang, Se-Yeong Park, Seung-Hwan Lee, Apri Heri Iswanto, Nam Hun Kim

**Affiliations:** 1 Department of Forest Biomaterials Engineering, College of Forest and Environmental Sciences, Kangwon National University, Chuncheon, 24341 Republic of Korea; 2 Institute of Forest Science, Kangwon National University, Chuncheon, 24341 Republic of Korea; 3 Department of Forest Products Technology, Faculty of Forestry, FC Korea Land Co., Ltd., Seoul, 07271 Republic of Korea; 4 Universitas Sumatera Utara, Medan, 20155 Indonesia

**Keywords:** Boiling water treatment, Dimensional and color changes, Moisture adsorption, *Quercus suber* reproduction cork, *Quercus variabilis* virgin cork, Volume shrinkage, Water absorption, Weight loss, Engineering, Materials science

## Abstract

The effects of boiling water treatment on the physical properties of *Quercus variabilis* virgin cork (*Qv* VC) were examined and compared with those of *Quercus suber* reproduction cork (*Qs* RC). The water treatment was conducted at 100 °C for 1 h. *Qv* VC showed a significantly higher dimensional change in the three directions and lower weight loss than *Qs* RC by boiling water treatment. Untreated and boiled *Qv* VC showed higher density, air-dried moisture content, red/green (a*) and yellow/blue (b*) chromaticity, overall color change, shrinkage in all three directions, moisture adsorption on the entire surface, and swelling per 1% moisture content than untreated and boiled *Qs* RC. However, the lightness (L*) and water absorption on each surface were higher for *Qs* RC than for *Qv* VC. Moisture adsorption on each surface was comparable before and after heat treatment for both species. After boiling water treatment, the air-dried moisture content, dimensions, volume shrinkage, water absorption, and moisture adsorption on each surface and the entire surface increased, whereas L*, a*, b*, and swelling per 1% moisture content decreased. The results of the present study could be useful for further utilization of *Qv* cork growing in Korea.

## Introduction

Cork from the outer bark of oak species is a natural, renewable, and sustainable raw material because of properties such as light weight, excellent thermal and sound insulation, impermeability to liquids and gases, superior shock absorption, high coefficient of friction, and high resistance to microbial activity^1–3^. Three types of cork are exploited from cork oak trees: virgin cork (VC), second cork, and reproduction cork (RC). VC and second cork with uneven tissue structures are typically used for the trituration of agglomerate products. RC is the most important material for producing solid cork products because of its uniform structure^4^.

*Quercus suber* (*Qs*) is a well-established cork resource for the cork industry worldwide^5^, and it is mainly found in Mediterranean countries such as Algeria, France, Italy, Morocco, Portugal, Spain, and Tunisia^6^. *Qs* RC consists of 91–95% cork cells and 5–8% lenticular channels^7^.

*Quercus variabilis* (*Qv*) is one of the most widespread tree species in East Asia, particularly in China, Japan, and Korea^8^. *Qv* VC consists of 84–87% cork cells, 11–13% lenticular channels, and 2–3% dark-brown zones and sclereids^7^. Because of its structural characteristics, *Qv* cork is commonly used for the trituration of cork granules and agglomeration to produce cork composite products. In China, *Qv* cork is cultivated, exploited, and used on a limited scale for cork production^9^.

In the cork industry, boiling water treatment for 1 h is a common and well-established method for increasing the flexibility of cork, flattening cork planks, removing dirt from cork, and improving its dimensional expansion and mechanical properties^4,10,11^. Several studies have been conducted on the effects of boiling water treatment on the physical properties of *Qs* RC. Rosa et al*.*^10^ reported that the boiling treatment of *Qs* RC reduced cork cell-wall corrugations, resulting in an expansion of cork dimensions by 10–15% in the radial direction and 5–7% in the tangential and longitudinal directions. Rosa and Fortes^12^ reported that the maximum volume swelling of *Qs* RC cork after being immersed in water at 20 °C for approximately 70 h was 4.4%, with a moisture content of 58%. In contrast, the maximum volume swelling of *Qs* cork boiled at 100 °C for 28 h was 7.8%, with a moisture content of 200%. In addition, the diffusion coefficients of water in *Qs* RC at 90 °C were two-fold higher than those in *Qs* RC at 20 °C.

Cumbre et al*.*^13^ reported that boiling *Qs* RC in water resulted in the expansion of all growth rings in the radial direction and decreased the density, pore number, and pore area. The boiling water treatment also caused radial failure in the lenticular channel of *Qs* RC.

Few studies have investigated the effects of boiling water treatment on the physical properties of *Qv* cork. Yuan et al*.*^14^ reported that boiling water treatment of *Qv* VC and RC grown in China resulted in the attenuation of cell wall corrugation, increased cork volume, and decreased cork density. The brightness of both corks decreased after the boiling water treatment, whereas the color difference increased as the treatment duration increased. Song et al*.*^11^ reported that boiling water treatment at approximately 100 °C for *Qv* RC decreased the density, lightness, red/green chromaticity, and yellow/blue chromaticity as the heat treatment duration increased, whereas the water absorption and total color differences gradually increased with the increase in boiling time.

As mentioned in previous studies^7,15^, the cork industry in Korea is seeking alternative cork resources from domestic forests to reduce the import of cork material from Europe and China. *Qv* VC is the only cork resource currently available in Korea, and a comprehensive study of the utilization of VC is needed. Therefore, in this study, the effects of boiling water treatment on the physical properties of Korean *Qv* VC were investigated and compared with those of commercial *Qs* RC to provide valuable information for the further utilization of *Qv* cork grown in Korea.

## Methods

### Materials

**Figure 1:**
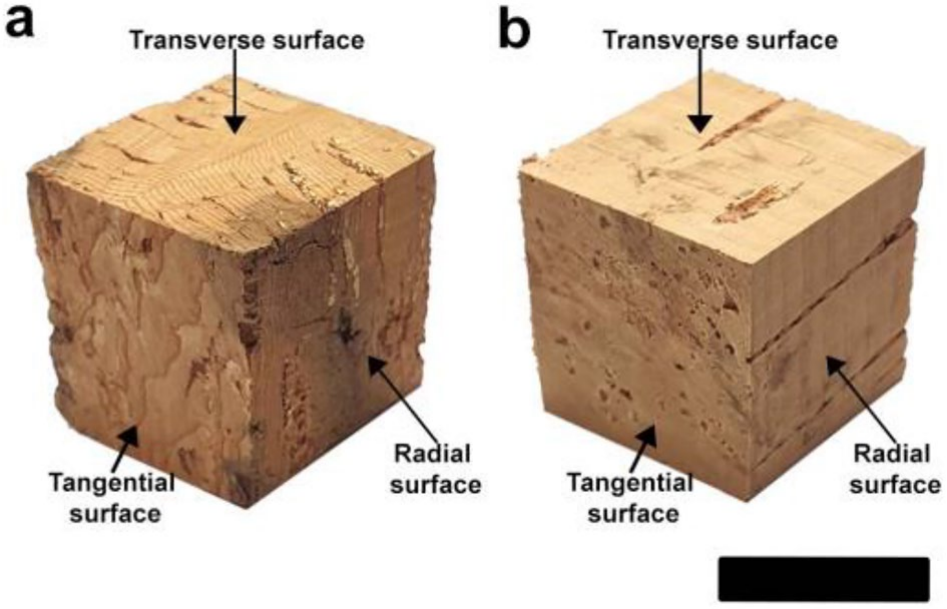
Cork cube samples with dimensions of 20 mm (R) × 20 mm (T) × 20 mm (L): *Qv* VC (**a**) and *Qs* RC (**b**).

In this study, we examined previously used samples^7,15^, which was collected from three trees at breast height of *Qv* VC in the research forest of Kangwon National University, Chuncheon, Korea (37° 77′ N, 127° 81′ E). Two planks of *Qs* RC from the Castelo Branco cork forest of Amorim Group (Mozelos, Portugal) were provided by FC Korea Land Co., Ltd., Seoul, Korea. The cork samples were obtained in compliance with all institutional, national, and international guidelines and legislation. The essential information on the experimental corks is listed in Table 1. The *Qv* VC and *Qs* RC samples (Fig. 1) with dimensions of 20 mm (radial, R) × 20 mm (tangential, T) × 20 mm (longitudinal, L) were oven-dried and kept in a conditioning room at 25 ± 5 °C and 60 ± 5% relative humidity (RH).

**Table 1:** Essential information about the cork samples.

Species	Cork type	Cork thickness (mm)	Growth ring numbers	Growth ring width (mm)	Green density (g/cm^3^)	Green moisture content (%)
*Qv*	VC	10–30	48–52	0.43–0.61	0.39 (0.09)	19.3 (1.7)
*Qs*	RC	30–40	10–11	3.05–3.08	–	–

Numbers within parentheses are standard deviations.

### Methods

#### Boiling treatment process

The boiling treatment was performed using tap water as the heating medium. Water (500 mL) was boiled in a 2000 mL glass beaker by using a hotplate (GLMS-C12; Global Lab, Siheung-si, Korea). The target temperature of the water, approximately 100 °C, was measured using an infrared thermometer (Raynger Mx Series; Raytek, Santa Cruz, USA). The cork samples were thoroughly soaked in boiling water for 1 h^4^. The samples were removed from the glass beaker after naturally reaching 30 ± 5 °C and subsequently air-dried in the laboratory atmosphere at 25 ± 5 °C and 60 ± 5% RH. During the drying process, boiled cork samples of both species were weighed daily until a constant weight was achieved.

### Evaluation of anatomical characteristics before and after boiling water treatment

#### Macroscopic observation

To observe the changes in the macroscopic characteristics of the transverse, radial, and tangential surfaces before and after boiling, air-dried cork samples with dimensions of 20 mm (R) × 20 mm (T) × 20 mm (L) from each species were prepared and trimmed using a sliding microtome (MSL-H; Nippon Optical Works, Nagano, Japan) before boiling water treatment. Images of the three surfaces were obtained before and after the boiling water treatment by using a mobile phone and recorded in a digital format of 3024 × 3024 pixels with a resolution of 72 dpi and a depth of 24 bits (Samsung Note 20, 12MP with F1.8; Suwon-si, Korea).

#### Scanning electron microscopy

To perform scanning electron microscopy of the transverse, radial, and tangential surfaces of both untreated and boiled corks, air-dried cork samples with dimensions of 10 mm (R) × 10 mm (T) × 10 mm (L) from each species were prepared and trimmed using a sliding microtome (MSL-H model; Nippon Optical Works). The samples were coated with gold by using a sputter coater (Cressington Sputter Coater 108; Watford, UK) and observed under a scanning electron microscope (SEM; JEOL, JSM-5510, 15 kV, Tokyo, Japan).

### Measurement of physical properties before and after boiling water treatment

Detailed information regarding the physical properties of both cork samples is listed in Table 2.

**Table 2:** Sample information for evaluating the physical properties of heat-treated cork.

Test	Sample dimension	Species	Treatment	Sample number*	Total
Color change	20 mm (R) × 20 mm (T) × 20 mm (L)	*Qv* VC and *Qs* RC	Boiled	10^R^ × 2^Sp^ × 1^T^	20
Moisture content	20 mm (R) × 20 mm (T) × 20 mm (L)	*Qv* VC and *Qs* RC	Untreated and boiled	10^R^ × 2^Sp^ × 2^T^	40
Density	20 mm (R) × 20 mm (T) × 20 mm (L)	*Qv* VC and *Qs* RC	Untreated and boiled	10^R^ × 2^Sp^ × 2^T^	40
Weight loss	20 mm (R) × 20 mm (T) × 20 mm (L)	*Qv* VC and *Qs* RC	Boiled	10^R^ × 2^Sp^ × 1^T^	20
Dimensional change after boiling water treatment	20 mm (R) × 20 mm (T) × 20 mm (L)	*Qv* VC and *Qs* RC	Boiled	10^R^ × 2^Sp^ × 1^T^	20
Shrinkage from green to oven-dry	20 mm (R) × 20 mm (T) × 20 mm (L)	*Qv* VC and *Qs* RC	Untreated and boiled	10^R^ × 2^Sp^ × 2^T^	40
Water absorption	20 mm (R) × 20 mm (T) × 20 mm (L)	*Qv* VC and *Qs* RC	Untreated and boiled	10^R^ × 2^Sp^ × 2^T^ × 3^S^	120
Moisture adsorption on each surface	20 mm (R) × 20 mm (T) × 20 mm (L)	*Qv* VC and *Qs* RC	Untreated and boiled	10^R^ × 2^Sp^ × 2^T^ × 3^S^	120
Moisture adsorption on the entire surface	20 mm (R) × 20 mm (T) × 5 mm (L)	*Qv* VC and *Qs* RC	Untreated and boiled	10^R^ × 2^Sp^ × 2^T^	40

Sample number = replication (^R^) × species (^Sp^) × treatment (^T^) × surface (^S^).

#### Color change

Color was evaluated before and after boiling water treatment by using a chromameter (CR-10 Plus; Konica Minolta, Tokyo, Japan). Ten air-dried cork samples with dimensions of 20 mm (R) × 20 mm (T) × 20 mm (L) from each species were prepared. The color of the transverse, radial, and tangential surfaces of the untreated and boiled corks was determined using the CIE L*a*b* system, which is characterized by three parameters: L* (lightness), a* (red/green chromaticity), and b* (yellow/blue chromaticity). The overall color change was calculated using Eq. (1).

1 $$\Delta E* = \left( {\Delta L^{*2} + \Delta a^{*2} + \Delta b^{*2} } \right)^{\frac{1}{2}}$$

where ΔL*, Δa*, Δb*, and ΔE* are changes in lightness, red/green chromaticity, yellow/blue chromaticity, and overall color, respectively.

#### Moisture content and density

To measure moisture content and density, twenty air-dried cork samples with dimensions of 20 mm (R) × 20 mm (T) × 20 mm (L) from each species were prepared from the untreated and boiled corks. The moisture content and density after air drying were determined according to Korean standards KS F 2199^16^ and KS F 2198^17^ by using Eqs. (2) and (3), respectively.

2 $$M = \left( { \frac{{M_{a} - M_{o} }}{{M_{o} }}} \right) \times 100 \left( \% \right)$$

where *M* (%) is the moisture content of air-dried cork samples, *M*_*a*_ (g) is the weight of air-dried cork samples, and *M*_*o*_ (g) is the weight of oven-dried cork samples.

3 $$D_{a} = { }\frac{{M_{a} }}{{V_{a} }} \left( {{\text{g}}/{\text{cm}}^{3} } \right)$$

where *D*_*a*_ (g/cm^3^) is the density of air-dried cork samples, *M*_*a*_ (g) is the weight of air-dried cork samples, and *V*_*a*_ (cm^3^) is the volume of air-dried cork samples.

#### Weight loss

In this study, the weight loss of ten oven-dried samples with dimensions of 20 mm (R) × 20 mm (T) × 20 mm (L) from each cork species was measured to understand the effects of boiling water treatment by using Eq. (4).

4 $$WL = \left( { \frac{{m_{1} - m_{2} }}{{m_{1} }}} \right) \times 100 \left( \% \right)$$

where *WL* (%) is the weight loss, *m*_*1*_ (g) is the weight of the oven-dried samples before boiling water treatment, and *m*_*2*_ (g) is the weight of the oven-dried samples after boiling water treatment.

#### Dimensional change after boiling water treatment

Dimensional changes after boiling water treatment in the radial (*L*_*r*_), tangential (*L*_*t*_), and longitudinal *(L*_*l*_) directions were calculated using Eq. (5). Ten air-dried cork samples with dimensions of 20 mm (R) × 20 mm (T) × 20 mm (L) from each species were used to determine dimensional changes after boiling water treatment. Volumetric changes were calculated as the sum of the radial, tangential, and longitudinal changes.

5 $$L_{{\left( {r, t, l} \right) }} = \left( { \frac{{L_{2} - L_{1} }}{{L_{1} }}} \right) \times 100 \left( \% \right)$$

where *L* (%) is the dimensional change in each direction of the air-dried samples before (*L*_*1*_*,* mm) and after (*L*_*2*_, mm) boiling water treatment.

#### Shrinkage

Shrinkage from green to oven-dried in the radial, tangential, and longitudinal directions of the untreated and heat-treated samples was determined according to Korean standard KS F 2203^18^ by using Eq. (6). Untreated and heat-treated twenty oven-dried cork samples with dimensions of 20 mm (R) × 20 mm (T) × 20 mm (L) were prepared for each species. The cork samples were immersed in distilled water at 25 ± 1 °C, and length was measured in the radial (*L*_*r*_), tangential (*L*_*t*_), and longitudinal (*L*_*l*_) directions at intervals of 3 days until the dimensions in the directions remained constant. We define these samples as green corks; subsequently, the green cork samples were oven-dried at 100 °C for 24 h. Volumetric shrinkage was obtained by adding the radial, tangential, and longitudinal shrinkages.

6 $$S_{o} = \left( { \frac{{l_{g} - l_{o} }}{{l_{o} }}} \right) \times 100 \left( \% \right)$$

where *S*_*o*_ (%) is the total shrinkage in each direction from green to oven-dry conditions, and *l*_*g*_ (mm) and *l*_*o*_ (mm) are the lengths in the three directions under green (more than 100% moisture content) and oven-dried conditions, respectively.

#### Water absorption

Water absorption on each surface of the untreated and treated samples was determined according to Korean standard KS F 2204^19^ by using Eq. (7). Sixty oven-dried cork samples with dimensions of 20 mm (R) × 20 mm (T) × 20 mm (L) were prepared for each cork species. The oven-dried cork samples coated with paraffin, except for the measuring surface, were soaked in distilled water at 25 ± 1 °C for 24 h.

7 $$W_{ab} = \left( { \frac{{W_{2} - W_{1} }}{A}} \right) \left( {{\text{g}}/{\text{cm}}^{2} } \right)$$

where *W*_*ab*_ (g/cm^2^) is water absorption, *W*_*1*_ (g) is the weight before soaking the samples, *W*_*2*_ (g) is the weight after soaking the samples, and *A* (cm^2^) is the absorption area.

#### Moisture adsorption on each surface

Moisture adsorption on each surface of the untreated and treated samples was determined according to Korean standard KS F 2205^20^. Sixty oven-dried cork samples with dimensions of 20 mm (R) × 20 mm (T) × 20 mm (L) were prepared for each species to determine moisture adsorption on the transverse, radial, and tangential surfaces of untreated and boiled corks. The cork samples with constant weight at 75 ± 2% RH and 40 ± 1 °C in a Thermo hygrostat (TH3-PE; JEIO TECH, Daejeon, Korea) were coated with paraffin, except for the measuring surface. Subsequently, the samples were placed in a chamber at 90 ± 2% RH and 40 ± 1 °C for 24 h. Moisture adsorption was calculated using Eq. (8).

8 $$M_{ad} = \left( { \frac{{W_{24h} - W_{oh} }}{A}} \right) \left( {{\text{g}}/{\text{cm}}^{2} } \right)$$

where *M*_*ad*_ is moisture adsorption (g/cm^2^), *W*_*0h*_ (g) is the weight stabilized at 75% RH and 40 °C, *W*_*24h*_ (g) is the weight after placing the samples in a desiccator at 90% RH and 40 °C for 24 h, and *A* (cm^2^) is the adsorption area.

#### Moisture adsorption on the entire surface

Moisture adsorption on the entire surface of both untreated and treated samples was conducted according to the methodology of Jo et al.^21^ and Korean standard KS F 2205^20^. Twenty oven-dried cork samples with dimensions of 20 mm (R) × 20 mm (T) × 5 mm (L) were prepared for each species. The constant weight of the cork samples at 75 ± 2% RH and 40 ± 1 °C and 90 ± 2% RH and 40 ± 1 °C was obtained in the Thermo hygrostat. Moisture adsorption (equilibrium moisture content [EMC]) and swelling per 1% moisture content were calculated using Eq. (9).

$${\text{EMC at RH }}75{\text{\% }} = \left( { \frac{{W_{{\left( {oh} \right)}} - W}}{W}} \right) \times 100 \left( \% \right)$$

$${\text{EMC at RH }}90{\text{\% }} = \left( { \frac{{W_{\left( \infty \right)} - W}}{W}} \right) \times 100 \left( \% \right)$$

9 $${\text{Swelling}}\;{\text{per}}\;{1}\% \;{\text{moisture}}\;{\text{content}} = \left( { \frac{{L_{{\left( {t,r} \right)}} - l_{{\left( {t,r} \right)}} }}{{l_{{\left( {t,r} \right)}} }}} \right) \times \left( { \frac{W}{{W_{\left( \infty \right)} - W_{{\left( {oh} \right)}} }}} \right)\left( {\text{\% }} \right)$$

where *W* (g) is the oven-dried weight, and *W*_*0h*_ (g) and *l* (mm) are the weight and length stabilized at 75% RH and 40 °C, respectively. *W*_*∞*_ (g) and *L* (mm) are the weight and length stabilized at 90% RH and 40 °C, respectively.

#### Statistical analysis

Statistical differences in air-dried moisture content and density, weight loss, dimensional and color changes, water absorption, and moisture adsorption between treatments and species were analyzed using one-way analysis of variance (ANOVA) followed by post-hoc Tukey’s honestly significant difference (HSD) test (SPSS ver. 24, IBM Corp., New York, USA).

## Results and discussion

### Macroscopic characteristics

**Figure 2:**
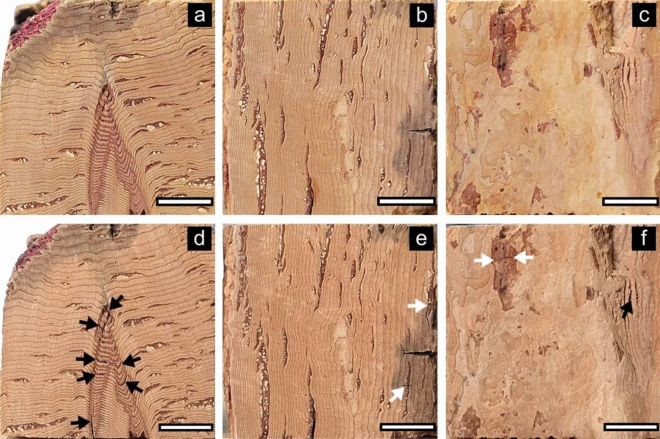
Transverse (**a**,**d**), radial (**b**,**e**), and tangential (**c**,**f**) surfaces before (**a**–**c**) and after (**d**–**f**) boiling water treatment in *Qv* VC. Black and white arrows indicate the cracks in LFT (**d**,**f**) and DBZ (**e**,**f**) after boiling water treatment, respectively.

**Figure 3:**
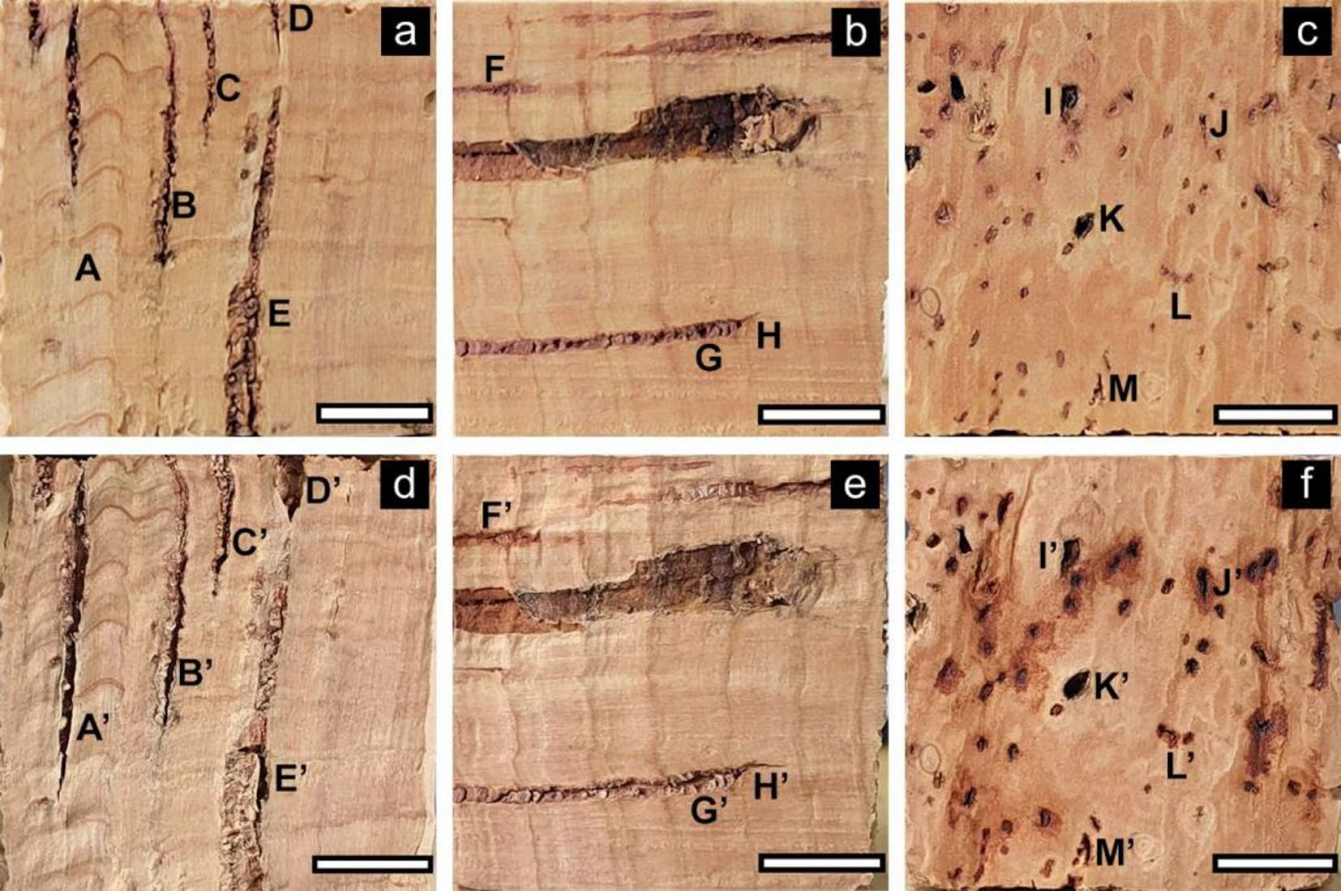
Transverse (**a**,**d**), radial (**b**,**e**), and tangential (**c**,**f**) surfaces before (**a**–**c**) and after (**d**–**f**) boiling water treatment in *Qs* RC. A–M in (**a**–**c**) and A’–M’ in (**d**–**f**) indicate the changes in the lenticular channel of *Qs* RC after boiling water treatment, respectively.

Appearances of the transverse, radial, and tangential surfaces of *Qv* VC and *Qs* RC before and after boiling water treatment are shown in Figs. 2 and 3, respectively. *Qv* VC changed to a darker color after boiling, whereas *Qs* RC changed to a pale color. In addition, the tangential surface of *Qs* RC exhibited a darker coloration around the lenticular channel. Some cracks were found across the cork cells, dark-brown zone (DBZ), and lenticular filling tissue (LFT) of *Qv* VC after the water boiling treatment. In contrast, no cracks were detected in the lenticular channels of *Qs* RC, with an enlarged shape and darkening after the water boiling treatment (for example, K → K’ and J → J’).

A few studies support our results regarding the effects of boiling water treatment on macroscopic structural changes. Song et al*.*^11^ reported that *Qv* cork changed to a darker color after boiling water treatment at approximately 100 °C for 3 h. Cumbre et al*.*^13^ reported that boiling water treatment of *Qs* RC caused enlargement of lenticular channels in the tangential direction.

### Microscopic characteristics

**Figure 4:**
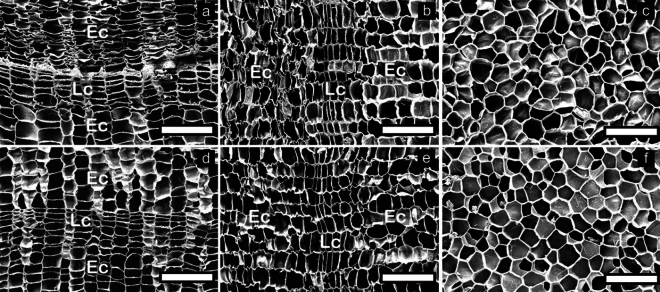
Transverse (**a**,**d**), radial (**b**,**e**), and tangential (**c**,**f**) surfaces before (**a**–**c**) and after (**d**–**f**) boiling water treatment in *Qv* VC. Earlycork (Ec) and latecork (Lc).

**Figure 5:**
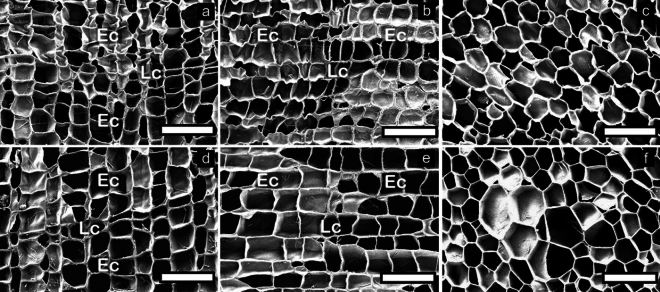
Transverse (**a**,**d**), radial (**b**,**e**), and tangential (**c**,**f**) surfaces before (**a**–**c**) and after (**d**–**f**) boiling water treatment in *Qs* RC. Earlycork (Ec) and latecork (Lc).

SEM images of the transverse, radial, and tangential surfaces of *Qv* VC and *Qs* RC before and after boiling water treatment are shown in Figs. 4 and 5, respectively. The boiling water treatment resulted in the straightening of the corrugated cork cell wall in *Qv* VC and *Qs* RC, especially in earlycork cells. Rosa et al*.*^10^ reported that the boiling treatment of *Qs* RC resulted in the straightening of the cork cell wall, particularly pronounced in the corrugated earlycork cells. Yuan et al*.*^14^ reported that the boiling treatment of *Qv* VC reduced cork cell wall corrugations.

**Figure 6:**
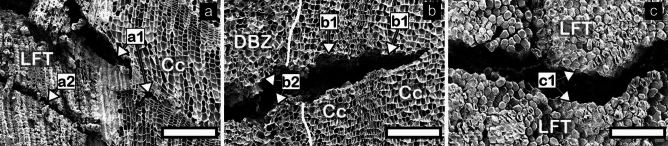
Transverse (**a**), radial (**b**), and tangential (**c**) surfaces after boiling water treatment in *Qv* VC. Cc, LFT, and DBZ indicate cork cells, lenticular filling tissue, and dark brown zone, respectively. Cracks in compact LFT (a2, c1), cork cells (a1, b1), and DBZ (b2). The white solid line (**b**) indicates the boundary between cork cells and DBZ.

Figure 6 shows SEM images of cracks in the cork samples treated with boiling water. In *Qv* VC, cracks parallel to the growth rings were observed in cork cells adjacent to the LFT (Fig. 6a, a1) and within LFT (Fig. 6a, a2) on the transverse surface. Cracks perpendicular to the growth rings were observed in cork cells (Fig. 6b, b1) and DBZ (Fig. 6b, b2) on the radial surface. Cracks were also observed within compact LFT cells on the tangential surface (Fig. 6c, c1).

### Color change

Color changes according to the CIE L*a*b* system on the transverse, radial, and tangential surfaces of *Qv* VC and *Qs* RC before and after the boiling water treatment are presented in Tables 3 and 4.

**Table 3:** L*, a*, and b* changes of *Qv* VC and *Qs* RC before and after boiling water treatment.

Species	Treatment	L*			a*			b*		
		Transverse	Radial	Tangential	Transverse	Radial	Tangential	Transverse	Radial	Tangential
*Qv* VC	Untreated	44.51^bA^ (0.53)	43.44^bA^ (0.51)	50.74^cB^ (0.52)	16.84^cC^ (0.23)	15.92^dB^ (0.22)	14.66^cA^ (0.24)	23.93^cB^ (0.25)	24.26^cB^ (0.25)	22.79^cA^ (0.21)
	Boiled	40.12^aA^ (0.37)	39.93^aA^ (0.24)	44.83^aB^ (0.47)	10.30^bB^ (0.23)	10.28^cB^ (0.25)	9.49^bA^ (0.16)	19.03^aB^ (0.22)	19.62^aC^ (0.26)	18.68^aA^ (0.18)
*Qs* RC	Untreated	52.43^dA^ (0.39)	52.43^dA^ (0.36)	54.07^dB^ (0.42)	10.08^bB^ (0.24)	9.45^bA^ (0.25)	9.35^bA^ (0.11)	22.49^bB^ (0.29)	22.54^bB^ (0.28)	21.93^bA^ (0.18)
	Boiled	47.66^cB^ (0.34)	48.17^cB^ (0.37)	46.67^bA^ (0.37)	8.77^aA^ (0.28)	8.49^aA^ (0.31)	8.40^aA^ (0.29)	18.67^aA^ (0.21)	19.20^aB^ (0.21)	18.67^aA^ (0.25)

Numbers within parentheses represent standard deviations. Numbers in the same column with the same superscript lowercase letters indicate non-significant outcomes at 5% significance level for comparisons between treatments and species. The mean value in the same line followed by the same capital indicates non-significant outcomes at 5% significance level for comparisons between surfaces.

**Table 4:** ∆E* of *Qv* VC and *Qs* RC after boiling water treatment.

Species	∆E*		
	Transverse	Radial	Tangential
*Qv* VC	8.79^bB^ (0.25)	8.11^bA^ (0.17)	8.72^bB^ (0.13)
*Qs* RC	6.47^aA^ (0.41)	6.42^aA^ (0.40)	7.65^aB^ (0.26)

Numbers within parentheses represent standard deviations. Numbers in the same column with the same superscript lowercase letters indicate non-significant outcomes at 5% significance level for comparisons between treatments and species. The mean value in the same line followed by the same capital indicates non-significant outcomes at 5% significance level for comparisons between surfaces.

The L* values on the three surfaces of both corks decreased significantly after boiling water treatment. Untreated and boiled *Qs* RC had significantly higher L* values than untreated and boiled *Qv* VC.

The L* values on the tangential surface of untreated and boiled *Qv* VC were significantly higher than those on the transverse and radial surfaces, whereas no significant differences were found in the L* values between the transverse and radial surfaces of the untreated and boiled samples. In contrast, the L* values of untreated *Qs* RC were significantly higher on the tangential surface than on the transverse and radial surfaces, whereas those of boiled *Qs* RC were significantly higher on the transverse and radial surfaces than on the tangential surface. No significant differences were observed in the L* values of untreated and boiled *Qs* RC between the transverse and radial surfaces.

On all three surfaces of both cork species, the a* values significantly decreased after boiling water treatment. The three surfaces of untreated and boiled *Qv* VC exhibited significantly higher a* values than those of untreated and boiled *Qs* RC. In untreated *Qv* VC, the a* values were the highest on the transverse surface and lowest on the tangential surfaces, and significant differences were detected between the three surfaces. In boiled *Qv* VC, a* values were significantly higher on the transverse and radial surfaces than on the tangential surface, and the transverse and radial surfaces showed comparable a* values. In untreated *Qs* RC, the a* values on the transverse surface was significantly higher than those on the radial and tangential surfaces, and no significant difference was observed between the radial and tangential surfaces. For boiled *Qs* RC, no differences in the a* values were found among the three surfaces.

In both cork species, the b* values of the three surfaces significantly decreased after the boiling water treatment. Untreated and boiled *Qv* VC showed higher b* values on the three surfaces than untreated and boiled *Qs* RC. Significant differences were found in the b* values between untreated *Qv* VC and *Qs* RC, whereas no significant differences were observed in the b* values between boiled corks of either species. The b* values of untreated and boiled *Qv* VC were significantly lower on the tangential surface than on the transverse and radial surfaces. In untreated *Qv* VC, no significant differences were found in b* values between the transverse and radial surfaces, whereas b* values on the radial surface of boiled *Qv* VC were significantly higher than those on the transverse surface. For untreated *Qs* RC, b* values were significantly lower on the tangential surface than on the transverse and radial surfaces, whereas the transverse and radial surfaces had comparable values. In boiled *Qs* RC, b* values were significantly higher on the radial surface than on both transverse and tangential surfaces. No significant differences in b* values were observed between the radial and tangential surfaces.

In *Qv* VC, ∆E* values on the radial surface were significantly lower than those on the transverse and tangential surfaces. No significant differences in ∆E* values were observed between the transverse and tangential surfaces. In *Qs* RC, ∆E* values were significantly higher on the tangential surface than on the transverse and radial surfaces. No significant differences in ∆E* values were found between the transverse and radial surfaces. *Qv* VC showed significantly higher ∆E* values on each surface than *Qs* RC.

In this study, L*, a*, and b* values of both cork species significantly decreased after boiling water treatment, which is consistent with the results of Yuan et al*.*^14^ and Song et al*.*^11^. Yuan et al*.*^14^ reported that the L* values of *Qv* cork decreased after boiling water treatment and the color differences increased as the duration time increased. Song et al*.*^11^ reported that boiling water treatment decreased the L*, a*, and b* values of *Qv* cork and the ∆E* of *Qv* cork gradually increased with as the boiling time increased. The L*, a*, and b* values of untreated *Qs* RC reported in this study were lower than those of *Qs* cork reported by Pereira^4^.

The decrease in L*, a*, and b* values of cork after boiling water treatment could be related to the extraction of phenolic compounds, such as tannins. As mentioned by Rosa et al*.*^10^ and Pereira^4^, boiling water treatment could solubilize mostly phenolic compounds, such as tannins, from cork, corresponding to the brown color of the water during the boiling process. Until now, there is limited information on the color change of cork by boiling water treatment. Nevertheless, a few studies have been reported on the relationship between extractives and color change in wood, which supports our results. Moura and Brito^22^ reported that the a* value of *Eucalyptus grandis* wood decreased after heat treatment, which could be related to the volatilization of certain chemical compounds, such as phenolic extractives. Tolvaj et al.^23^ found that the woods of *Pinus sylvestris* and *Picea abies* became darker after steam treatment, and the color hue of both species turned brown. The observed color change in both species could be attributed to the alteration of the conjugated double bond systems found mainly in extractives. A high amount of extractives in a wood species resulted in significant color changes during steaming^24,25^.

### Moisture content and density

The air-dried moisture content and density of boiled *Qv* VC and *Qs* RC samples are listed in Table 5. The moisture content and density of untreated and boiled *Qv* VC were significantly higher than those of *Qs* RC. The moisture content of both *Qv* and *Qs* corks increased after boiling water treatment. A significant difference in moisture content was observed between untreated and boiled *Qv* VC, whereas no significant differences in moisture content were found between untreated and boiled *Qs* RC. In addition, the densities of both *Qv* and *Qs* corks did not change before and after boiling water treatment.

**Table 5:** Moisture content and density of *Qv* VC and *Qs* RC before and after boiling water treatment.

Species	Treatment	Air-dried moisture content (%)	Density (g/cm^3^)
*Qv* VC	Untreated	5.47^b^ (0.33)	0.22^b^ (0.02)
	Boiled	6.16^c^ (0.29)	0.21^b^ (0.02)
*Qs* RC	Untreated	4.61^a^ (0.34)	0.17^a^ (0.01)
	Boiled	5.08^ab^ (0.36)	0.16^a^ (0.01)

Numbers within parentheses represent standard deviations. Numbers in the same column with the same superscript lowercase letters indicate non-significant outcomes at 5% significance level for comparisons between treatments and species.

The results of previous studies are consistent with those of this study. Rosa et al*.*^10^ reported that the density of *Qs* RC after boiling water treatment gradually decreased as the boiling duration increased. Yuan et al*.*^14^ reported that the density of both *Qv* VC and *Qv* RC decreased after boiling water treatment, with a ratio of 0.85–0.88 for VC and 0.79–0.80 for RC. Song et al*.*^11^ reported that the density of *Qv* RC after boiling water treatment decreased as the treatment duration increased, with a ratio range of 0.79–0.82. The authors also mentioned that the decrease in density was correlated with reduced cork mass and increased cork dimensions during the boiling water treatment.

### Weight loss and dimensional change

Table 6 shows the weight loss and dimensional changes in boiled *Qv* VC and *Qs* RC. The weight loss after water boiling treatment for *Qv* VC and *Qs* RC was 2.20% and 2.67%, respectively, showing a significant difference between the two species.

**Table 6:** Weight loss and dimensional change of *Qv* VC and *Qs* RC after boiling water treatment.

Species	Weight loss (%)	Dimensional change (%)			
		Radial	Tangential	Longitudinal	Volume
*Qv* VC	2.20^a^ (0.36)	6.94^bC^ (0.26)	0.86^bA^ (0.14)	1.75^bB^ (0.01)	9.56^b^ (0.27)
*Qs* RC	2.67^b^ (0.46)	3.58^aC^ (0.19)	0.23^aA^ (0.14)	0.49^aB^ (0.14)	4.30^a^ (0.13)

Numbers within parentheses represent standard deviations. Numbers in the same column with the same superscript lowercase letters indicate non-significant outcomes at 5% significance level for comparisons between species. The mean value in the same line followed by the same capital indicates non-significant outcomes at 5% significance level for comparisons between directions.

Both corks swelled in the radial, tangential, and longitudinal directions after the water boiling treatment, and dimensional expansion was the highest in the radial direction and lowest in the tangential direction. A significant difference was observed in the dimensional expansion between each direction. Moreover, *Qv* VC exhibited significantly higher swelling in all three directions and volumes than *Qs* RC.

The results of previous studies on *Qs* and *Qv* corks are consistent with those of this study. Rosa et al*.*^10^ reported that the volume expansion of boiled *Qs* RC was 10–15% in the radial direction and 5–7% in the tangential and longitudinal directions. They mentioned that the expansion in the radial direction was entirely due to the attenuation of the corrugations in the lateral cell walls, which become straightening in the radial direction. Cumbre et al*.*^13^ found that the growth ring width of boiled *Qs* RC increased by 12.8% at the 1st growth ring and 26.9% at the 8th growth ring, where the 1st growth ring had a smaller width than the 8th growth ring. Yuan et al*.*^14^ reported that the maximum volume expansion of boiled *Qv* cork was 15.4–17.1% for VC and 24.8–26.8% for RC.

Song et al*.*^11^ reported that the dimensional change in *Qv* RC after boiling water treatment was higher in the radial direction than in the non-radial direction. *Qv* RC after boiling water treatment showed volume expansion and softening and internal stress release of the cork, resulting in increasing the cork flexibility. In addition, boiling water treatment caused the partial straightening of the lateral cell walls, particularly in the radial direction. The authors also reported that the decreased mass of the cork sample after boiling water treatment corresponded to the dissolution of some phenolic compounds and water-soluble lignin.

**Figure 7:**
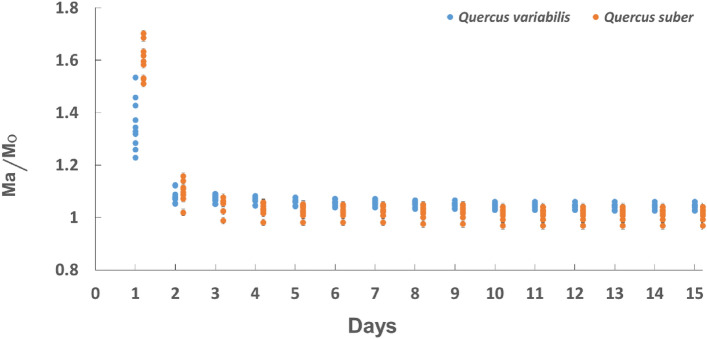
Air-drying curves of *Qv* VC and *Qs* RC (Ma/Mo: mass of boiled samples/oven-dry mass before boiling.

Air-drying curves in the laboratory atmosphere at 25 ± 5 °C and 60 ± 5% RH for *Qv* VC and *Qs* RC after the water boiling treatment are shown in Fig. 7. Mass change during air-drying for both *Qv* VC and *Qs* RC rapidly decreased from day 1 to day 2, followed by a gradual decrease from day 2 to day 10, and it finally became constant from day 10 to day 15. Rosa et al*.*^10^ also reported that mass change during the air-drying of *Qs* RC rapidly decreased from day 1 to day 2, followed by a gradual decrease from day 2 to day 6, and it then became constant from day 6 to day 8.

### Shrinkage

Shrinkage from green to oven-dry for *Qv* VC and *Qs* RC before and after the boiling water treatment is presented in Table 7. Shrinkage in the three directions of boiled *Qv* VC and *Qs* RC was significantly higher than that of untreated samples. In addition, the volumetric shrinkage of both corks significantly increased after the boiling water treatment. Untreated and boiled *Qv* VC showed significantly higher shrinkage in all three directions and volumes than untreated and boiled *Qs* RC. In untreated and boiled corks of both species, shrinkage was the highest in the radial direction and lowest in the longitudinal direction. A significant difference in shrinkage was observed among the three directions for each species. The anisotropy coefficients of untreated and boiled *Qv* VC and *Qs* RC were comparable, and no significant differences in the anisotropy coefficients of each condition were observed between the two species.

**Table 7:** Shrinkage from green to oven-dry of *Qv* VC and *Qs* RC before and after boiling water treatment.

Species	Treatment	Radial	Tangential	Longitudinal	Volumetric	T/R
*Shrinkage* (%)						
*Qv* VC	Untreated	2.32^cC^ (0.15)	1.72^cB^ (0.10)	1.35^cA^ (0.14)	5.40^c^ (0.23)	0.74^a^ (0.07)
	Boiled	2.59^dC^ (0.14)	1.92^dB^ (0.09)	1.51^dA^ (0.11)	6.02^d^ (0.20)	0.74^a^ (0.05)
*Qs* RC	Untreated	1.58^aC^ (0.13)	1.28^aB^ (0.10)	0.78^aA^ (0.14)	3.59^a^ (0.23)	0.81^a^ (0.08)
	Boiled	1.86^bC^ (0.09)	1.47^bB^ (0.09)	0.97^bA^ (0.06)	4.29^b^ (0.16)	0.79^a^ (0.06)

Numbers within parentheses represent standard deviations. Numbers in the same column with the same superscript lowercase letters indicate non-significant outcomes at 5% significance level for comparisons between treatments and species. The mean value in the same line followed by the same capital indicates non-significant outcomes at 5% significance level for comparisons between directions.

Kim et al*.*^26^ reported that the shrinkage in *Qv* RC was the highest in the radial direction and lowest in the longitudinal direction, whereas that in *Qs* RC was the highest in the tangential direction and lowest in the radial direction. They also mentioned that shrinkage in the radial and tangential directions in *Qv* RC was significantly greater than that in *Qs* RC, whereas shrinkage in the longitudinal direction was significantly greater in *Qs* RC than in *Qv* RC. Pereira^4^ reported that the cork of *Qs* with 400–500% moisture content or immersed cork with moisture content more than 200% in hot water for long periods and then subjected to oven-drying at 100 °C showed significant volume shrinkage of up to 30%.

In the present study, the shrinkage of both corks was the highest in the radial direction, showing comparable results with swelling properties. As reported in some studies^10^, cork commonly showed a noticeable change in the radial direction by drying or boiling water treatment, due to partial straightening or wrinkling of the cell lateral walls in the radial direction.

### Water absorption

Water absorption on each surface before and after boiling water treatment is presented in Table 8. For both *Qv* VC and *Qs* RC, water absorption on the transverse, radial, and tangential surfaces significantly increased after boiling water treatment. For both untreated and boiled *Qv* VC, there was no significant difference in water absorption between the transverse, radial, and tangential surfaces. In contrast, for both untreated and boiled *Qs* RC, the tangential surface showed significantly greater water absorption than the transverse and radial surfaces, with no significant differences in water absorption between the transverse and radial surfaces.

**Table 8:** Water absorption of *Qv* VC and *Qs* RC before and after boiling water treatment.

Species	Treatment	Transverse	Radial	Tangential
*Water absorption* (g/cm^2^)				
*Qv* VC	Untreated	0.013^bA^ (0.002)	0.013^bA^ (0.001)	0.012^aA^ (0.002)
	Boiled	0.017^cA^ (0.002)	0.018^cA^ (0.003)	0.018^bA^ (0.002)
*Qs* RC	Untreated	0.010^aA^ (0.002)	0.010^aA^ (0.002)	0.016^bB^ (0.002)
	Boiled	0.022^dA^ (0.002)	0.023^dA^ (0.002)	0.038^cB^ (0.003)

Numbers within parentheses represent standard deviations. Numbers in the same column with the same superscript lowercase letters indicate non-significant outcomes at 5% significance level for comparisons between treatments and species. The mean value in the same line followed by the same capital indicates non-significant outcomes at 5% significance level for comparisons between surfaces.

Rosa and Fortes^12^ reported that water absorption on the tangential surface at 20 °C and 90 °C was significantly higher and faster than that on the transverse and radial surfaces. They also mentioned that water absorption on the transverse surface was slightly higher than that on the radial surface and water absorption increased as the temperature increased.

The transverse and radial surfaces of untreated *Qv* VC showed significantly higher water absorption than those of untreated *Qs* RC, whereas the tangential surface of untreated *Qs* RC showed significantly higher water absorption than that of untreated *Qv* VC. After boiling water treatment, *Qs* RC showed significantly higher water absorption on all three surfaces than *Qv* VC.

*Qv* VC consists of cork cells, lenticular filling tissue, DBZ, and sclereids^7,15^. Water absorption in cork could be related to cork properties, such as composition, arrangement, and porosity of cork elements. As mentioned by Prasetia et al*.*^15^, the lenticular channels of *Qs* RC have an opening with loose lenticular filling tissue, whereas *Qv* VC is filled with compact lenticular filling tissue. In addition, lenticular channels in *Qs* RC are abundant on the tangential surface. Pereira^4^ also explained that the lenticular filling tissue is more hygroscopic than cork cells because of higher polysaccharide levels and lack of suberin content, which could increase the contact area for water adsorption.

### Moisture adsorption on each surface

Moisture adsorption on each surface before and after the boiling water treatment is presented in Table 9. Moisture adsorption on each surface and total moisture adsorption in *Qv* VC slightly increased after boiling water treatment. However, no significant differences were observed between the untreated and boiled corks. Moisture adsorption of untreated and boiled *Qv* VC was comparable for the three surfaces. No significant differences were found in either untreated or boiled *Qv* VC among the three surfaces.

**Table 9:** Moisture adsorption on the three surfaces of *Qv* VC and *Qs* RC before and after boiling water treatment.

Species	Treatment	Transverse	Radial	Tangential	Total	B/U
*Moisture adsorption* (g/cm^2^)						
*Qv* VC	Untreated	0.008^abA^ (0.003)	0.007^abA^ (0.003)	0.009^aA^ (0.002)	0.025^ab^ (0.004)	1.18^a^ (0.24)
	Boiled	0.009^abA^ (0.002)	0.010^bcA^ (0.003)	0.010^aA^ (0.002)	0.029^bc^ (0.002)	
*Qs* RC	Untreated	0.006^aA^ (0.002)	0.005^aA^ (0.000)	0.010^aB^ (0.000)	0.021^a^ (0.002)	1.49^b^ (0.19)
	Boiled	0.011^bA^ (0.003)	0.011^cA^ (0.003)	0.011^aA^ (0.003)	0.031^c^ (0.002)	

B and U indicate boiled and untreated corks, respectively. Numbers within parentheses represent standard deviations. Numbers in the same column with the same superscript lowercase letters indicate non-significant outcomes at 5% significance level for comparisons between treatments and species. The mean value in the same line followed by the same capital indicates non-significant outcomes at 5% significance level for comparisons between surfaces.

In untreated *Qs* RC, the tangential surface exhibited significantly higher moisture adsorption than the transverse and radial surfaces, whereas no significant differences were observed between the transverse and radial surfaces. After boiling water treatment, moisture adsorption on the transverse and radial surfaces showed a significant increase, whereas on the tangential surface, there was no significant difference between untreated and boiled corks. Furthermore, the three surfaces of boiled *Qs* RC exhibited comparable moisture adsorption. The total moisture adsorption of untreated cork in *Qs* RC was significantly lower than that of boiled cork.

Moisture adsorption on the transverse and radial surfaces of untreated *Qv* VC was slightly higher than that of untreated *Qs* RC. In contrast, moisture adsorption on the tangential surface of untreated *Qv* VC was slightly lower than that of untreated *Qs* RC. Total moisture adsorption in untreated *Qv* VC was slightly higher than that in untreated *Qs* RC. No significant differences in moisture adsorption were found on the three surfaces between the untreated corks of both species. After boiling water treatment, *Qs* RC showed slightly higher moisture adsorption on the transverse, radial, and tangential surfaces than *Qv* VC. No significant differences in moisture adsorption were observed on the three surfaces between boiled corks of both species. In addition, the ratio of moisture adsorption in untreated and boiled *Qs* RC was significantly higher than that in *Qv* VC.

### Moisture adsorption on the entire surface

EMC and swelling per 1% moisture content of the corks before and after boiling treatment are presented in Table 10. Untreated and boiled corks of both species showed a significant increase in EMC with the increase in RH. In both corks, EMC at 75% and 90% RH significantly increased after the boiling water treatment. The EMC ratios (B/U) of untreated and boiled corks of both species were higher at 75% RH than at 90% RH. No significant differences were found in the EMC ratio of untreated and boiled corks between RH and species. Untreated and boiled *Qv* VC showed slightly higher EMC at 75% and 90% RH than untreated and boiled *Qs* RC. No significant differences were observed in the EMC of both species between untreated corks and between boiled corks.

**Table 10:** EMC and swelling per 1% moisture content of *Qv* VC and *Qs* RC before and after boiling water treatment.

Species	Treatment	EMC at 40 °C (%)		Swelling per 1% moisture content (%)		
		RH 75%	RH 90%	Radial	Tangential	T/R
*Qv* VC	Untreated	4.97^abA^ (0.45)	7.15^abB^ (0.55)	0.020^bB^ (0.002)	0.018^bA^ (0.002)	0.90^b^ (0.01)
	Boiled	6.16^cA^ (0.23)	8.26^cB^ (0.22)	0.015^aB^ (0.002)	0.012^aA^ (0.002)	0.81^a^ (0.01)
	B/U	1.23^aA^ (0.12)	1.16^aA^ (0.07)	0.77^aB^ (0.06)	0.64^aA^ (0.06)	–
*Qs* RC	Untreated	4.38^aA^ (0.43)	6.32^aB^ (0.16)	0.019^bB^ (0.004)	0.017^bA^ (0.004)	0.89^b^ (0.01)
	Boiled	5.49^bcA^ (0.27)	7.49^bcB^ (0.55)	0.014^aB^ (0.001)	0.011^aA^ (0.001)	0.78^a^ (0.01)
	B/U	1.25^aA^ (0.11)	1.18^aA^ (0.11)	0.75^aB^ (0.06)	0.63^aA^ (0.08)	−

B and U indicate boiled and untreated cork, respectively, and T/R is the anisotropy coefficient. Numbers within parentheses represent standard deviations. Numbers in the same column with the same superscript lowercase letters indicate non-significant outcomes at 5% significance level for comparisons between treatments and species. The mean value in the same line followed by the same capital indicates non-significant outcomes at 5% significance level for comparison between RH level and direction.

Gonzales-Adrados and Haro^27^ reported that the moisture adsorption of water-boiled *Qs* RC at 20 °C and 40 °C constantly increased from 20 RH to 80% RH and significantly increased from 80 RH to 90% RH. The ratio (m/m_0_: mass at 90% RH/initial mass) of moisture adsorption at 90% RH was approximately 1.12 at 20 °C and 1.10 at 40 °C, respectively. Moisture adsorption was lower at 40 °C than at 20 °C, with a decrease of approximately 2–3% between the two temperatures. Pereira^4^ reported that the EMC of *Qs* cork at ambient temperatures of approximately 15–25 °C was 8% at 75% RH, 10% at 85% RH, and 16% at 95% RH. When compared with the EMC of other hygroscopic materials such as wood, the EMC of cork is relatively low: 15.5% to 19.5% at 20 °C and 90% RH in six Argentine wood species^28^, 20% at ambient temperatures and 80% RH in cork oak wood^4^, and 9.6% at 75% RH and 13.9% at 90% RH and 40 °C in Paulownia wood^16^.

The increase in water adsorption of cork after boiling water treatment can be attributed to the expansion of cork cell dimensions, extraction of extractives, and failures such as cracks and abnormal deformation in the cell components of cork. As mentioned by Pereira et al*.*^29^ and Pereira^4^, boiling water treatment extracts small amounts of water-soluble extractives from cork. Rosa et al*.*^10^ and Yuan et al*.*^14^ reported that boiling water treatment of *Qs* RC and *Qv* VC reduced cork cell wall corrugations.

The swelling per 1% moisture content and anisotropy coefficient of both boiled cork species were significantly lower than those of the untreated samples. No significant differences in swelling per 1% moisture content and the anisotropy coefficients were observed before and after boiling treatment in both species. Moreover, in both species, swelling per 1% moisture content and the ratio (B/U) before and after boiling treatment were significantly higher in the radial direction than in the tangential direction. The reduced swelling of boiled cork may be due to the straightening of cell walls during the boiling water treatment. Several studies^10,11,13,14^ have reported the positive effects of boiling water treatment on volume expansion of corks. Pereira^4^ reported that a second boiling water treatment of boiled cork did not cause additional swelling.

## Conclusions

- The boiling water treatment of *Qv* VC and *Qs* RC resulted in straightening of the corrugated cork cell wall and cracks in the LFT, cork cells, and DBZ of *Qv* VC as well as enlargement of lenticular channels in the tangential direction of *Qs* RC.
- The L*, a*, and b* values on all three surfaces of both *Qv* VC and *Qs* RC decreased significantly after boiling water treatment. The ∆E* values on the three surfaces of *Qv* VC were significantly higher than those on the three surfaces of *Qs* RC.
- The air-dried moisture content, dimensional change, shrinkage in the three directions, and volume of *Qv* VC and *Qs* RC increased with the boiling water treatment, whereas the density was constant. *Qv* VC showed a significantly greater dimensional change after boiling water treatment than *Qs* RC, whereas *Qs* RC showed a significantly greater weight loss than *Qv* VC. Untreated and boiled *Qv* VC showed significantly higher shrinkage in the three directions and volume than untreated and boiled *Qs* RC.
- After boiling water treatment, water absorption on the three surfaces of *Qv* VC and *Qs* RC significantly increased, and *Qs* RC showed significantly higher water absorption on all three surfaces than *Qv* VC.
- Moisture adsorption on the transverse, radial, and tangential surfaces of *Qv* VC and *Qs* RC increased after the boiling water treatment. After treatment, *Qs* RC showed slightly higher moisture adsorption on the three surfaces than *Qv* VC.
- EMC at 75% RH and 90% RH in both *Qv* VC and *Qs* RC significantly increased after boiling water treatment, whereas the dimensional change per 1% moisture content significantly decreased. Untreated and boiled *Qv* VC showed a higher EMC at 75% and 90% RH and swelling per 1% moisture content than untreated and boiled *Qs* RC.

In conclusion, the effects of boiling water treatment on the physical properties of *Qv* VC grown in Korea, such as cork cell wall straightening, increasing shrinkage, water absorption, moisture adsorption, and decreasing L*, a*, and b* values, were revealed. The results can be used to understand *Qv* VC quality and provide valuable information for the further utilization of the cork. Owing to the anatomical structure and physical properties, the *Qv* VC could be potentially use as cork granules and agglomeration for composite products.

## Data Availability

The datasets generated and analyzed during the current study are not publicly available but are available from the corresponding author on reasonable request.
