# Author Correction: Effects of boiling water treatment on the physical properties of *Quercus variabilis* virgin cork grown in Korea

**DOI:** 10.1038/s41598-024-61091-6

**Published:** 2024-05-14

**Authors:** Denni Prasetia, Byantara Darsan Purusatama, Jong Ho Kim, Jae Hyuk Jang, Se‑Yeong Park, Seung‑Hwan Lee, Apri Heri Iswanto, Nam Hun Kim

**Affiliations:** 1 Department of Forest Biomaterials Engineering, College of Forest and Environmental Sciences, Kangwon National University, Chuncheon, 24341 Republic of Korea; 2 Institute of Forest Science, Kangwon National University, Chuncheon, 24341 Republic of Korea; 3 Department of Forest Products Technology, Faculty of Forestry, FC Korea Land Co., Ltd., Seoul, 07271 Republic of Korea; 4 Universitas Sumatera Utara, Medan, 20155 Indonesia

Correction to: *Scientific Reports* 10.1038/s41598-024-56110-5, published online 05 March 2024

The original version of this Article contained errors in the Reference list.

Reference 7 was incorrectly given as:

Prasetia, D. et al*.* Quantitative anatomical characteristics of VC in *Quercus variabilis* grown in Korea. *Forests* **13**, 1711. 10.3390/f13101711 (2022).

The correct reference is listed below:

Prasetia, D. et al*.* Quantitative anatomical characteristics of Virgin Cork in *Quercus variabilis* grown in Korea. *Forests* **13**, 1711. 10.3390/f13101711 (2022).

In addition, Reference 9 was incorrectly given as:

Ferreira, J., Miranda, I., Şen, U. & Pereira, H. Chemical and cellular features of chemical and cellular features of virgin and RC from *Quercus variabilis*. *Ind. Crops Prod.* **94**, 638–648 (2016).

The correct reference is listed below:

Ferreira, J., Miranda, I., Şen, U. & Pereira, H. Chemical and cellular features of chemical and cellular features of virgin and reproduction cork from *Quercus variabilis*. *Ind. Crops Prod.* **94**, 638–648 (2016).

Finally, Reference 26 was incorrectly given as:

Kim, B. R., Mishiro, A., Sugitama, J. & Okano, T. The physical properties of virgin and RCs of *Quercus variabilis* Blume. *Bull. Tokyo Univ. For.* **82**, 199–217.

The correct reference is listed below:

Kim, B. R., Mishiro, A., Sugiyama, J. & Okano, T. The physical properties of virgin and reproduction corks of *Quercus variabilis* Blume. *Bull. Tokyo Univ. For.* **82**, 199–217.

The original Article has been corrected.

